# Targeted genetic testing for familial hypercholesterolaemia using next generation sequencing: a population-based study

**DOI:** 10.1186/1471-2350-15-70

**Published:** 2014-06-23

**Authors:** Penny J Norsworthy, Jana Vandrovcova, Ellen RA Thomas, Archie Campbell, Shona M Kerr, Jennifer Biggs, Laurence Game, Anne K Soutar, Blair H Smith, Anna F Dominiczak, David J Porteous, Andrew D Morris, Generation Scotland, Timothy J Aitman

**Affiliations:** 1 MRC Clinical Sciences Centre, Faculty of Medicine, Imperial College London, London, UK; 2 Institute of Genetics and Molecular Medicine, University of Edinburgh, Crewe Road South, Edinburgh EH4 2XU, UK; 3 Medical Research Institute, University of Dundee, Dundee, UK; 4 College of Medical, Veterinary and Life Sciences, University of Glasgow, Glasgow, UK; 5 Generation Scotland, A Collaboration between the University Medical Schools and NHS in Aberdeen, Dundee, Edinburgh and Glasgow, UK

**Keywords:** Familial hypercholesterolaemia, Total cholesterol, *LDLR*, Molecular diagnostic testing, Next-generation sequencing, Primary care, Generation Scotland

## Abstract

**Background:**

Familial hypercholesterolaemia (FH) is a common Mendelian condition which, untreated, results in premature coronary heart disease. An estimated 88% of FH cases are undiagnosed in the UK. We previously validated a method for FH mutation detection in a lipid clinic population using next generation sequencing (NGS), but this did not address the challenge of identifying index cases in primary care where most undiagnosed patients receive healthcare. Here, we evaluate the targeted use of NGS as a potential route to diagnosis of FH in a primary care population subset selected for hypercholesterolaemia.

**Methods:**

We used microfluidics-based PCR amplification coupled with NGS and multiplex ligation-dependent probe amplification (MLPA) to detect mutations in *LDLR*, *APOB* and *PCSK9* in three phenotypic groups within the Generation Scotland: Scottish Family Health Study including 193 individuals with high total cholesterol, 232 with moderately high total cholesterol despite cholesterol-lowering therapy, and 192 normocholesterolaemic controls.

**Results:**

Pathogenic mutations were found in 2.1% of hypercholesterolaemic individuals, in 2.2% of subjects on cholesterol-lowering therapy and in 42% of their available first-degree relatives. In addition, variants of uncertain clinical significance (VUCS) were detected in 1.4% of the hypercholesterolaemic and cholesterol-lowering therapy groups. No pathogenic variants or VUCS were detected in controls.

**Conclusions:**

We demonstrated that population-based genetic testing using these protocols is able to deliver definitive molecular diagnoses of FH in individuals with high cholesterol or on cholesterol-lowering therapy. The lower cost and labour associated with NGS-based testing may increase the attractiveness of a population-based approach to FH detection compared to genetic testing with conventional sequencing. This could provide one route to increasing the present low percentage of FH cases with a genetic diagnosis.

## Background

Familial hypercholesterolaemia (FH, OMIM no.143890) is a common autosomal dominant disorder characterised by raised serum cholesterol, increased arterial deposition of low density lipoprotein (LDL)-cholesterol and high risk of premature coronary heart disease (CHD). Clinical diagnosis is based on the presence of raised total or LDL-cholesterol, tendon xanthomata, corneal arcus, family history and the presence of FH-causing mutations in the *LDLR*, *PCSK9* or *APOB* genes [1]. The prevalence of FH is estimated to be between 1/200 and 1/500 [2,3], and while most of the excess risk of premature CHD can be averted by cholesterol lowering drugs, currently only 12-25% of cases in the UK, and <1% in the USA are believed to have a molecular diagnosis [3,4].

Genetic testing and family cascade screening has been proposed as the most cost-effective strategy for detecting cases of FH [3,5], and for distinguishing monogenic FH from sporadic or polygenic hypercholesterolaemia [6]. This strategy was adopted in the 2008 UK guidelines from the National Institute for Health and Clinical Excellence (NICE) [7]. However, implementation of these guidelines in England has been limited due to poor availability, low throughput and high costs of traditional genetic testing, fragmented service delivery and lack of investment in identification of index cases.

We previously demonstrated that next generation sequencing (NGS) is suitable for FH mutation detection in patients attending lipid clinics, with similar sensitivity and specificity to conventional capillary sequencing [8]. Moreover, NGS mutation detection is suited to high-throughput testing of large numbers of samples, which is not so readily achievable with standard capillary sequencing platforms. Since the majority of undiagnosed FH patients receive their healthcare outside lipid clinics, we sought to assess the feasibility of targeted sequence-based genetic testing for FH in a primary care-based population subset selected for hypercholesterolaemia.

## Methods

### DNA samples

The Generation Scotland: Scottish Family Health Study (GS:SFHS) is a repository of anonymised biological samples and accompanying clinical, biochemical and family data from 23,960 Scottish residents [9]. The ability to recruit at least one first-degree relative was a condition for participation in the GS:SFHS, and therefore around two thirds of those taking part are consenting adult relatives of recruits and volunteers.

**Figure 1:**
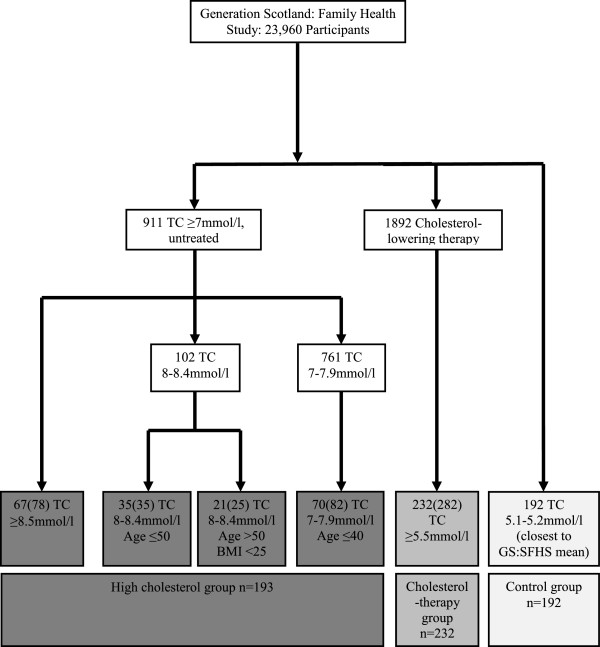
**Selection criteria for GS:SFHS high cholesterol, cholesterol-therapy and control groups**. Groups of subjects selected for the study are shaded. The number of unrelated participants studied in each category of total cholesterol level, age and body mass index is shown. Numbers in parentheses denote the total number of participants in the entire GS:SFHS cohort within each study group. TC: Total cholesterol.

We selected a subset of GS:SFHS subjects for genetic analysis using criteria designed to enrich the test population for individuals likely to have FH, based on previously reported population total cholesterol data in FH [10,11], and using additional age and body mass index (BMI) thresholds to reduce inclusion of age- and obesity-related cases of hyperlipidaemia (Figure 1). Triglyceride levels were not measured in the Generation Scotland cohort and therefore data for LDL-cholesterol were not available. Subjects were consequently selected for study on the basis of total cholesterol data. In total, 617 unrelated subjects were obtained from the GS:SFHS and passed quality control (QC; see below) for NGS and MLPA: 193 individuals with raised total cholesterol (designated high cholesterol group), 232 individuals with moderately high total cholesterol despite cholesterol-lowering therapy (cholesterol-therapy group) and 192 normocholesterolaemic controls. The control group, also from the GS:SFHS study, were selected to be closest to the population specific mean total cholesterol level, and were age, sex and BMI matched to the high cholesterol group (Table 1 and Figure 1). Total cholesterol cut-off thresholds for each study group are defined in Figure 1. Of all study subjects, 84.1% were White Scottish, 8.6% other Caucasians, 2.0% mixed race and other ethnic groups, and 5.3% unknown ethnicity.

**Table 1:** Characteristics of Generation Scotland groups studied

**Group**	**Age**	**Sex distribution %M: %F**	**BMI**	**Total cholesterol (mmol/l)**
High cholesterol (n = 193)	44 (21–88)	41/59	26.9 (18.7-47.4)	8.1 (7.0-12.0)
Control (n = 192)	45 (21–84)	42/58	26.4 (14.2-44.6)	5.2 (5.0-5.3)
Cholesterol-therapy (n = 232)	60 (30–88)	43/57	28.1 (18.1-56.6)	5.9 (5.5-9.5)

Median data are given for age, BMI and total cholesterol. Ranges are shown in brackets.

Demographic data of the study population is summarised in Table 1. The median age of the cholesterol-therapy group was higher than the high cholesterol and control groups reflecting higher cholesterol-lowering drug prescription rates in older subjects in this primary care population. 40% of these individuals were known to be taking statins, while the remainder were on unspecified cholesterol lowering medication. Study subjects in the cholesterol-therapy group were selected from those with total cholesterol ≥5.5 mmol/l, these being unlikely to have achieved lipid-lowering therapy targets for FH [12]. DNA samples for available first-degree relatives of index cases who tested positive for FH mutations were obtained from the GS:SFHS repository, and were tested by Sanger sequencing for mutations detected in their respective proband relatives. Ethical approval for the GS:SFHS study was obtained from the Tayside Committee on Medical Research Ethics (on behalf of the National Health Service). All patients provided informed consent for the use of their data and samples for medical research.

### Mutation detection

The full *LDLR* coding sequence, all four exons known to harbour FH mutations in *PCSK9*, and the known mutation hot spot in *APOB* were amplified using the 48.48 Access Array (Fluidigm, San Francisco, USA) and pooled samples sequenced on a MiSeq sequencer (Illumina, San Diego, USA) as described [8]. Due to the very low likelihood of detecting mutations that could be definitively shown to be pathogenic in the remainder of the *PCSK9* and *APOB* genes, it was considered that inclusion of these regions in the assay would not be informative and these regions were therefore not included in the assay. In addition, because of the low frequency of known mutations outside coding regions or splice sites, intronic regions outside splice sites were not covered in this assay.

DNA sequences were considered to have passed QC if >90% of all nucleotides were covered at ≥15x and >98% of *LDLR* coding regions were covered at ≥15×. Mean coverage for all DNA samples passing QC across all amplicons was 2023× with a mean of 95.2% of bases covered at least 15×. Although this level of coverage falls below recent recommendations for clinical grade sequencing [13], our group established high sensitivity (98%) and specificity (100%) for this NGS-assay at comparable levels of coverage in a lipid clinic population [8]. Recent advances in MiSeq sequencing protocols and further assay optimisation would, however, allow further development that could lead to levels of coverage that meet present recommendations.

The presence of large insertions/deletions was detected using the MLPA (Multiplex Ligation-dependent Probe Amplification) *LDLR*-P062 kit (MRC-Holland, Amsterdam, The Netherlands) following the manufacturer’s protocol including recommendations for QC.

### Variant analysis

DNA sequences were mapped to the human GRCh37 reference assembly and variant calls generated and annotated as described [8]. Variants with minor allele frequency reported at greater than 1% in Ensembl [14], and intronic variants outside exon-intron boundaries were considered non-pathogenic and were excluded from further analysis. Remaining variants were compared to an FH locus-specific database [14,15], HGMD professional [16], and annotated *in silico* using Variant Effect Predictor [14] and Mutation Taster [17]. Variants described unequivocally as FH-causing in mutation databases were classified as pathogenic. All remaining known variants were classified as variants of uncertain clinical significance (VUCS) unless published data indicated that these variants are likely benign. Novel missense variants were also classified as VUCS. Novel sequence changes outside coding regions and essential splice sites, and synonymous coding changes were considered likely benign. As only 5’ and 3’ ends of introns were represented in our NGS-assay, intronic variants outside splice sites were not evaluated. All rare variants were verified by conventional Sanger sequencing and the single detected duplication was confirmed by PCR.

## Results

### Mutation detection

FH-causing mutations were detected in 4 out of 193 (2.1%) subjects in the high-cholesterol group and in 5 out of 232 (2.2%) subjects in the cholesterol-therapy group (Table 2). All nine mutations have been previously reported as pathogenic in FH patients [15,16]. No FH-causing mutations were detected in the 192 subjects in the normocholesterolaemic control group.

**Table 2:** Pathogenic FH mutations and variants of uncertain clinical significance (VUCS) found in Generation Scotland subjects

**No.**	**Age**	**Sex**	**Total cholesterol (mmol/l)**	**Group**	**Gene**	**Nucleotide change**	**Protein change**	**PolyPhen2 prediction**	**SIFT prediction**	**Mutation taster**	**n**
**Pathogenic mutations**											
1	51	F	12.0	High cholesterol	*APOB*	c.10580G > A	p.Arg3527Gln	Probably damaging	Deleterious	Polymorphism	1
2	44	F	8.9		*LDLR*	c.693C > G	p.Cys231Trp	Probably damaging	Deleterious	Disease causing	1
3	36	M	7.0		*LDLR*	c.268G > A	p.Asp90Asn	Probably damaging	Deleterious	Disease causing	1
4	61	F	8.3		*LDLR*	c.718G > A	p.Glu240Lys	Possibly damaging	Deleterious	Disease causing	1
5	56	M	5.9	Cholesterol-therapy	*APOB*	c.10580G > A	p.Arg3527Gln	Probably damaging	Deleterious	Polymorphism	1
6	57	M	5.7		*LDLR*	c.326G > A	p.Cys109Tyr	Probably damaging	Deleterious	Disease causing	1
7	50	M	5.9		*LDLR*	c.1133A > C	p.Gln378Pro	Probably damaging	Deleterious	Polymorphism	1
8	71	F	5.5		*LDLR*	c.232C > T	p.Arg78Cys	Probably damaging	Tolerated	Disease causing	1
9	60	F	6.2		*LDLR*	c.1586-?_1845 + ?dup		Not applicable	Not applicable	Not applicable	1
						(Exon 11 and 12 duplication)					
**VUCS**											
10	32	F	7.7	High cholesterol	*PCSK9*	c.274G > A^*a*^	p.Glu92Lys	Benign	Tolerated	Polymorphism	1
11	31	M	8.7		*LDLR*	c.-121 T > C	Not applicable	Not applicable	Not applicable	Polymorphism	1
12	54	F	8.2		*LDLR*	c.1816G > T	p.Ala606Ser	Possibly damaging	Tolerated	Disease causing	1
13	63	M	5.5	Cholesterol-therapy	*LDLR*	c.2294 T > G^*a*^	p.Val765Gly	Benign	Tolerated	Polymorphism	2^*b*^
14	61	M	7.0								
15	36	M	6.0		*LDLR*	c.2479G > A	p.Val827Ile	Probably damaging	Deleterious	Disease causing	1

n, number of individuals with variant, ^*a*^All variants had been reported previously apart from c.274G > A and c.2294 T > G which were novel variants, ^*b*^Not known to be related.

Five VUCS were detected in six subjects from the high-cholesterol and cholesterol-therapy groups (Table 2), but none in normocholesterolaemic controls. Three of the five detected variants have been previously reported in FH patients but their pathogenicity is unclear [8,15]. The two missense changes, p.Glu92Lys substitution in *PCSK9* and p.Val765Gly in *LDLR,* have not been reported before and further segregation and functional studies will be needed to clarify the pathogenicity of these variants.

Two additional rare non-synonymous coding sequence variants in *LDLR* were identified in three study subjects, but their previously reported presence in normocholesterolaemic individuals has led to their classification as benign variants [18]. The p.Arg744Gln variant was found in two cholesterol-treated subjects not known to be related, whose total cholesterol measurements on treatment were 7.7 mmol/l and 9.5 mmol/l. The p.Ala50Ser was found in one control subject (total cholesterol 5.3 mmol/l).

### Cascade testing

DNA was available for cascade testing in relatives of six of the index cases identified here as having pathogenic mutations. From a total of twelve available first-degree relatives, five molecular diagnoses of FH were made: one from the two relatives of index case 4, three from the five relatives of index case 8, and one from the single relative of index case 9 (Table 2). In all cases mutations identified in relatives were the same as that of the associated index case. Single first-degree relatives of index cases 2 and 5, and two first-degree relatives from index case 7, were mutation negative. Cholesterol data was available for three of the five new molecular diagnoses: two of these subjects had relatively high cholesterol levels despite being on cholesterol-lowering therapy (5.0 mmol/l and 5.5 mmol/l); the third subject was untreated, with a cholesterol level of 7.7 mmol/l.

## Discussion

Sequence-based detection of index cases and family cascade testing offer a possible route to diagnosis of FH in primary care, but this approach has not been evaluated to date. In this study we have used our recently validated NGS-based assay [8] and MLPA to detect FH-causing mutations in a primary care-based population in subjects who, based on readily available clinical data, were considered to have an increased risk of FH compared to the general population. In the sample of GS:SFHS patients tested, we found pathogenic, FH-causing mutations in 2.1% of unrelated high risk individuals and none in normocholesterolaemic controls. A further five FH cases were identified through cascade testing of first degree relatives of index cases by testing for the identified mutation found in each index case. We also detected VUCS in 1.4% of the high-cholesterol and cholesterol-therapy groups, but none in normocholesterolaemic controls. Further functional and segregation studies will be needed to clarify pathogenicity of these variants. The interpretation of VUCS in NGS projects remains a challenge, although given the present rapid increase in available, clinically relevant sequencing data and the expansion of disease-specific databases, we anticipate that the clinical interpretation of variants currently classified as VUCS is likely to become easier in the coming months and years.

The GS:SFHS is a primary care-based study of subjects aged 35–65 at recruitment, and their consenting adult relatives, phenotyped for a range of biochemical and morphometric parameters including body mass index, total cholesterol and history of medical prescriptions [9]. These clinical details were used to ascertain subjects who would be enriched for FH-causing mutations, but in most cases were not sufficient to apply formal clinical diagnostic criteria for FH (Simon Broome or Dutch Lipid Clinic) [19,20]. No data was collected on clinical phenotypes such as tendon xanthomata, and while some family history information was collected, it did not include sufficient detail (for example age at onset of cardiovascular disease) to apply Simon Broome or Dutch Lipid Clinic criteria. A family history of hypercholesterolaemia could only be established for those with relatives who had been recruited to the study. In subjects from the cholesterol-therapy group, pre-treatment cholesterol measurements were not available. Although preventing clinical diagnostic criteria from being applied, this level of information is analogous to the available data in a previous UK study of FH which used casenote review without genetic testing in a primary care setting [10]. This highlights the differences in entry criteria between primary care and Lipid Clinic populations, and illustrates the importance of careful selection criteria for this approach to molecular diagnosis in primary care. Of interest, however, was that within our high cholesterol group, a subset of 50 individuals who were aged 40 or under had total cholesterol 7–7.4 mmol/l and therefore do not meet Simon Broome criteria for FH. One of this group was found to have an FH causing mutation (see below).

The mutation detection rate reported here is considerably lower than in Lipid Clinic populations tested according to defined clinical criteria [8,21], but is higher than the detection rate in an unselected population study in Copenhagen, in which only 20% of those diagnosed clinically as having probable or definite FH (0.73% of the study population) were found by genotyping to have a common FH mutation [22]. The low FH detection rate in comparison to Lipid Clinic populations is most likely a consequence of the limited clinical data such as the absence of information on tendon xanthomata and LDL-cholesterol, as well as the probable inclusion of individuals with polygenic and lifestyle related hypercholesterolaemia which is reportedly of high prevalence in the Scottish compared to the English population [23] which may also have affected our ability to enrich this group for genetic effects. The availability of data on LDL-cholesterol and presence or absence of tendon xanthomata in future study populations, as well as genetic testing in populations with a lower background frequency of cardiovascular disease are likely to allow greater enrichment for patients with FH in those selected for testing, with a corresponding higher FH detection rate.

Treatment targets are more stringent in FH compared to sporadic hypercholesterolaemia owing to the greater vascular risk caused by lifelong high cholesterol levels [12], and therefore it is notable that the number of molecular diagnoses we made in the cholesterol-therapy group was similar to that of the high cholesterol group. Although individuals in the cholesterol-therapy group are receiving lipid-lowering medication, their relatively high cholesterol levels (5.5 – 6.2 mmol/l) suggest suboptimal treatment, which could most likely be addressed by more aggressive lipid-lowering therapy, ideally in a lipid clinic. Molecular diagnosis in these individuals would also permit cascade testing of their relatives. Consistent with the experience from the Dutch cascade screening programme [3,24], these data illustrate the advantages of identifying FH in primary care by genetic testing in order to distinguish FH patients definitively from those with other types of hypercholesterolaemia. Making use of NGS-based techniques to identify FH cases in primary care also provides a feasible alternative to reliance on lipid clinics in countries such as England and the USA where genetic testing for FH is not generally carried out and overall diagnostic rates remain low.

The selection criteria we employed here may not detect all mutation positive FH cases in primary care. For example, Index Case 3 (Table 1; TC = 7.0 mmol/l) was on the lower cholesterol threshold for inclusion in our study. In addition, since only 13% of patients in the GS:SFHS on cholesterol-lowering therapy had total cholesterol ≥5.5 mmol/l, further testing of subjects on cholesterol-lowering therapies but with cholesterol <5.5 mmol/l would be likely to make further molecular diagnoses of FH and these would be a source of additional cases through family cascade testing.

## Conclusions

To our knowledge, this is the first study of FH based on DNA sequencing in a primary care population. Previous studies to detect FH in primary care populations have been based on clinical criteria alone [10,25], or genotyping for common mutations [22]. We have evaluated the targeted use of high-throughput NGS-based methods for detection of FH cases in primary care, and demonstrated that population-based genetic testing using these protocols is able to deliver definitive molecular diagnoses of FH in high-risk individuals. Because detection of FH cases in primary care would require testing of much larger numbers of subjects than detection of cases in secondary care, the lower cost and labour associated with NGS-based testing [8,26,27] could increase the attractiveness of a population-based approach to FH detection compared to genetic testing with conventional sequencing. Coupled with genetic testing in lipid clinics, selective NGS-based population screening with family cascade testing could therefore be one route to increasing the present low percentage of FH cases with a genetic diagnosis. Detailed studies should now be undertaken to establish the feasibility and cost-effectiveness of this approach.

## Abbreviations

APOB: Apolipoprotein B; BMI: Body mass index; CHD: Coronary heart disease; FH: Familial hypercholesterolaemia; GS:SFHS: Generation Scotland: Scottish family health study; HGMD: Human gene mutation database; LDL: Low density lipoprotein; LDLR: Low density lipoprotein receptor; MLPA: Multiplex ligation-dependent probe amplification; NGS: Next generation sequencing; NICE: National institute for health and clinical excellence; PCSK9: Proprotein convertase subtilisin/kexin type 9; QC: Quality control; TC: Total cholesterol; VUCS: Variant of uncertain clinical significance.

## Competing interests

TJA and JV have a UK patent application pending on the NGS assay used in this report but have no plans to develop the patent. The remaining authors declare that they have no competing interests.

## Authors’ contributions

PJN carried out NGS assays, MLPA and compiled the clinical data. JV designed the NGS assay, oversaw data generation and analysed the NGS data. ERAT, JV, AKS, AC, SMK and TJA designed the selection criteria in Figure 1 and collated clinical information. AC and SMK supplied DNA samples and clinical data from the GS:SFHS resource. JB validated NGS data by capillary sequencing. LG oversaw generation of NGS MiSeq data. AKS participated in study design and preparation of the manuscript. BHS, AD, DP and AM conceived and oversee the GS:SFHS. TJA conceived and oversaw the study. PJN, JV and TJA co-wrote the manuscript. All authors read and approved the final manuscript.

## Pre-publication history

The pre-publication history for this paper can be accessed here:

http://www.biomedcentral.com/1471-2350/15/70/prepub
